# Vote of Thanks to Mr. Wakley by the Medical Profession of the City and County of Hereford

**Published:** 1844-11-02

**Authors:** 


### VOTE OF THANKS TO MR. WAKLEY BY THE MEDICAL PROFESSION OF THE CITY AND COUNTY OF HEREFORD.

To the Editor of the Lancet.

Sir,—I have the honour to convey to you the unanimous thanks of a public meeting of the medical practitioners of the city and county of Hereford, for the very able manner in which you have advocated the cause of the medical profession; and, in accordance with resolution 11, I herewith forward to you the proceedings of the meeting, and request, in its name, the favour of their insertion in your columns, at as early a period as may be convenient.

I remain, sir, your obedient servant,
Henry Graves Bull, Hon. Sec.

*Hereford*, Sept. 17, 1844.

[Mr. Wakley is the able Editor of the Lancet, and a member of Parliament.—Ed.]

